# Supplementary material for: Tuning myosin-driven sorting on cellular actin networks
Source: eLife. 2015 Mar 4;4:e05472. doi: 10.7554/eLife.05472 (PMC4377546; doi:10.7554/eLife.05472)
Supplement: Supplementary file 1. — Computer aided staple strand sequences for the flat-rectangular DNA origami scaffold. DOI: http://dx.doi.org/10.7554/eLife.05472.015 [file elife05472s001.docx]

**Supplementary file 1**

Computer aided staple strand sequences for the flat-rectangular DNA origami scaffold.

| **Core Strands** |  |
| --- | --- |
| r0t11ml1 | TTAGATACTATTTTCATTTGGGGAATGCCT |
| r0t11mr1 | TAAGAACGGAGGTTTTGAAGCCTAGTCAGA |
| r0t11mr2 | TAATGCAGTTCGAGCCAGTAATAACTGACCTA |
| r0t11mr3 | AAATCAGAGCTATTTTGCACCCAGAGAATAAC |
| r0t11mr_fr | ATAAGTCCATATTTAACAACGCCGTGTGAT |
| r0t11seam_l | AATATCGCTAAGAGGAAGCCCGAAACCTCCCG |
| r0t11seam_r | CCAGACGACGACAAAAGGTAAAGTATAACCTG |
| r0t13ml1 | GAGTAATGCGGAGACAGTCAAATAACGTTA |
| r0t13mr1 | TAAAGTACCGACAATAAACAACAGGTATTC |
| r0t13mr2 | AATTTAATAAATGCTGATGCAAATTTTTAATG |
| r0t13mr3 | GAGGCATTAACGCGCCTGTTTATCTTCATCGT |
| r0t13mr_fr | AAATAAGACCTTTTTAACCTCCGTGAGTGA |
| r0t13seam_l | TTTAGCTAATTTCGCAAATGGTCAAATTCTGT |
| r0t13seam_r | TATATTTTAGAACGCGAGAAAACTAAAGGGTG |
| r0t15mr1 | CAAGACAAAGTTAATTTCATCTTGAGAATA |
| r0t15mr3 | CTATATGTGGTTTGAAATACCGACCAACATGT |
| r0t15mr_fr | ATAACCTACAATAACGGATTCGTTATACTT |
| r0t15seam_l | AGAAAGGCTGTAGGTAAAGATTCATTTTCAAA |
| r0t17mr3 | TCAATTACACATAAATCAATATATGGCTTAGG |
| r0t19ml1 | CCACACAAGGGGTGCCTAATGAGAGCAGGC |
| r0t19ml3 | TCCTGTGTAATTGCGTTGCGCTCAAGAGAGTT |
| r0t19mr2 | CCCTCAATTAACACCGCCTGCAACATTCACCA |
| r0t19mr_fr | AACAGTTCCACCAGCAGAAGATACATTCTG |
| r0t19seam_r | AGCATCACGCCAGCAGCAAATGAAATAAAGTG |
| r0t1ml1 | TCCAAAAGTTTCGAGGTGAATTTGTAATGC |
| r0t1mr2 | TTTAACGGGAATGGAAAGCGCAGTCCATCTTT |
| r0t1seam_r | TTGATGATTCCAGTAAGCGTCATACGGTTTAT |
| r0t1t_seam | CGCCACCCTCAGAACCGCCACCCTCAGAACCG |
| r0t1tl1 | CCACCCTCAGAGCCACCACCCTCAAAAGGC |
| r0t1tl2 | GGATAGCAAGCCCAATAGGAACCCCAACAGTT |
| r0t1tl3 | TAACACTGAGTTTCGTCACCAGTTTTTCTGT |
| r0t1tr1 | GGTGTATCACCGTACTCAGGAGGTTAATAAGT |
| r0t21ml1 | GAAAATCCCCTTATAAATCAAAACGGCGAA |
| r0t21ml3 | AAGCGGTCGGTTGAGTGTTGTTCCGAGCCCCC |
| r0t21ml_fl | TTTCCAGTCGTAATCATGGTCACGAAAGGG |
| r0t21mr2 | GTCACACGTTGCAACAGGAAAAACTAAAGGGA |
| r0t21mr3 | GTCAGTATCAATATCTGGTCAGTTGCCCGAAC |
| r0t21mr_fr | GCCAACATGCTGGTAATATCCAAATCCTGA |
| r0t21seam_r | AAATGGATTACATTTTGACGCTCACGAAATCG |
| r0t23b_seam | CGTGGCGAGAAAGGAAGGGAAGAAAATCAGAG |
| r0t23bl1 | GATTTAGAGCTTGACGGGGAAAGCGAATAGCC |
| r0t23br1 | CGGGAGCTAAACAGGAGGCCGATGCTCATG |
| r0t23br2 | TTTTAGACAGGAACGGTACGCCAGGAACAATA |
| r0t23br3 | GAAGTGTTTTTATAATCAGTGAGCTCAAACT |
| r0t23ml2 | CGAGATAGCACGCTGGTTTGCCCCTGAGCTAA |
| r0t23ml_fl | ACAAGAGCACCGCCTGGCCCTGCTGCCCGC |
| r0t23mr1 | GAAATACCTATTTACATTGGCAGAGTGCCA |
| r0t23mr3 | GCCAGCCAACCAGTAATAAAAGGGAAAACAGA |
| r0t23seam_l | GCAAAATCTGTTTGATGGTGGTTCATCGTCTG |
| r0t3ml1 | CACTACGAATACACTAAAACACTATCTTGA |
| r0t3ml2 | GCTTGATATTGAAAATCTCCAAAAATTTTCAG |
| r0t3ml3 | GTTTCCATCGATTATACCAAGCGCGACCAGGC |
| r0t3ml_fl | ACAACCATTGCTAAACAACTTTATGTACCG |
| r0t3mr1 | TTTACCGTACAGGAGTGTACTGGTTAGTAC |
| r0t3mr2 | TCATAATCAATCAAGTTTGCCTTTCAAAAGGG |
| r0t3mr3 | TAAAGCCAGGTCAGTGCCTTGAGTGATATAAG |
| r0t3mr_fr | ACCGGAATCGATAGCAGCACCGGAAGGTAA |
| r0t3seam_l | CAGCTTGCGAGCCTTTAATTGTATCATGGCTT |
| r0t3seam_r | CATAGCCCCGCGTTTTCATCGGCACGAAAGAG |
| r0t5ml1 | CAAGAACCCTGCTCATTCAGTGAAATGCAG |
| r0t5ml2 | ACCCCCAGTAAACGGGTAAAATACCTTAAACA |
| r0t5ml_fl | GTACAACCTTTGAGGACTAAAGCAATGACA |
| r0t5mr1 | GACTGTAGCCTTATTAGCGTTTGCTCTGAA |
| r0t5mr2 | CGACATTCGAAACGCAAAGACACCATAATAAG |
| r0t5mr3 | AGCGACAGAAAATCACCGGAACCACAAACAAA |
| r0t5mr_fr | ATATTGAGCAAACGTAGAAAATAGCTATCT |
| r0t5seam_l | GCAAAAGAAGGCACCAACCTAAAATTTTCGGT |
| r0t5seam_r | ATATGGTTTTTGTCACAATCAATAAATCAACG |
| r0t7ml1 | ATACATAAAACACTATCATAACCTTGCATC |
| r0t7mr1 | AAGTTTATTACCAGCGCCAAAGAAGCGTCA |
| r0t7mr2 | AGCAAGAATGAACACCCTGAACAATAAATCAA |
| r0t7mr3 | ATATAAAAAACCGATTGAGGGAGGTAATCAGT |
| r0t7mr_fr | TACCGAAGCAGCCTTTACAGAGCTACAATT |
| r0t7seam_l | TAACAAAGGGATATTCATTACCCAGAAAATTC |
| r0t7seam_r | CCACAAGAGAGCGCTAATATCAGAGAGGCATA |
| r0t9ml1 | AAAAAGATGTTTTAATTCGAGCTTTGACCA |
| r0t9mr1 | GGGTAATTATTGAGTTAAGCCCAACGGAAT |
| r0t9mr2 | GATTAGTTTATAGAAGGCTTATCCTGTTCAGC |
| r0t9mr3 | GAATTAACACAATGAAATAGCAATACATACAT |
| r0t9mr_fr | TTATCCTCAAGCCGTTTTTATTAACAATAG |
| r0t9seam_l | GTAAGAGCCGCCAAAAGGAATTACGAGATAAC |
| r0t9seam_r | ACTTGCGGCGAGGCGTTTTAGCGAAGACTTCA |
| r1t0tr2 | GGATAAGTGCCGTCGAGAGGGTTAACAGTGC |
| r1t0tr3 | TAGGATTAGCGGGGTTTTGCTCAGTGCCTATT |
| r1t10fr1 | AGAACAAGGAATCTTACCAACGCTGTCAAAAA |
| r1t10fr2 | TCTTTCCATTAAACCAAGTACCGCATATCCCA |
| r1t10fr3 | AACGGGTAGAGCCTAATTTGCCAAATCCAA |
| r1t12fr1 | AGAATCGCCTGAACAAGAAAAATAACTCATCG |
| r1t12fr3 | TAAAGCCATACGAGCATGTAGAATTCCAAG |
| r1t14fr1 | GAGAGACTGCGTTAAATAAGAATACTTAATTG |
| r1t14fr2 | AATCATAATGAATTTATCAAAATCCGCTATTA |
| r1t14fr3 | GTCAATAGTTACTAGAAAAAGCCACCAGTA |
| r1t16fr1 | CGGGAGAATGCTTCTGTAAATCGTATAGGTCT |
| r1t16fr2 | ATTAATTTATACAGTAACAGTACCCTACCATA |
| r1t16fr3 | AGATGAATTCCCTTAGAATCCTTGAGAAGA |
| r1t18fr1 | CGACAACTATGGAAGGGTTAGAACTTTTACAT |
| r1t18fr2 | TCAAAATTAAGTATTAGACTTTACGGTTATCT |
| r1t18fr3 | GGATTTAGATTTGCACGTAAAACTAACGTC |
| r1t20fr1 | CGAACGAAGAAAGGAATTGAGGAAAAACAATT |
| r1t20fr2 | AAAATATCCTAAAACATCGCCATTGACCTGAA |
| r1t20fr3 | TGATAGCCTTTAGGAGCACTAACCATTTGA |
| r1t22fr1 | ATCGGCCTGAGATAGAACCCTTCTAAAAATAC |
| r1t22fr2 | AGCGTAAGTGCCTGAGTAGAAGAAGCCACCGA |
| r1t22fr3 | ACATCACTAATACGTGGCACAGACGCGAAC |
| r1t24br4 | GTAAAAGAGTCTGTCCATCACGCAGTAATA |
| r1t2fr1 | CAGACGATAACAGTTAATGCCCCCTACCAGGC |
| r1t2fr2 | TCGGAACCCATTGACAGGAGGTTGCGCCACCC |
| r1t2fr3 | GCCGCCAGTATTATTCTGAAACAGAAGGAT |
| r1t4fr1 | TGAAACCACCGCCTCCCTCAGAGCAGGCAGGT |
| r1t4fr2 | TCAGAACCTAGCAAGGCCGGAAACAAGGTGAA |
| r1t4fr3 | ATTACCATGCCACCCTCAGAGCCCAGAGCC |
| r1t6fr1 | GTATGTTACGGAAATTATTCATTAGTCACCAA |
| r1t6fr2 | TTATCACCATGATTAAGACTCCTTGTAAGCAG |
| r1t6fr3 | GAACTGGCGTCACCGACTTGAGCTAGCACC |
| r1t8fr1 | TGAAAATAGCCCTTTTTAAGAAAAATTACGCA |
| r1t8fr2 | ATAGCCGACGATTTTTTGTTTAACAACGAGCG |
| r1t8fr3 | ATAAGAAAACAAAGTTACCAGAACCCAAAA |
| r-1t0tl4 | CAACGCCTGTAGCATTCCACAGATTTGTCG |
| r-1t10fl1 | AAACGAGAGAGTACCTTTAATTGCTACGGTGT |
| r-1t10fl2 | ATAAGAGGCTCAAATGCTTTAAACAGAGGGGG |
| r-1t10fl3 | GAATCCCCTCATTTTTGCGGATGAGCTCAA |
| r-1t12fl2 | GGCAAAGAAAATATGCAACTAAAGTCCTTTTG |
| r-1t12fl3 | CATGTTTTATTAGCAAAATTAAGTTGTACC |
| r-1t14fl1 | GAGAAGCCGAGAGGGTAGCTATTTCATATGTA |
| r-1t14fl2 | TCTACAAATATGACCCTGTAATACACAGGCAA |
| r-1t14fl3 | AAAAACATGGCTATCAGGTCATTTGAACGG |
| r-1t16fl1 | CCCCGGTTGCTTTCATCAACATTACGTAACCG |
| r-1t16fl2 | CGAGTAACAAACTAGCATGTCAATTTGAGAGA |
| r-1t16fl3 | TAATCGTAAACCCGTCGGATTCTGGATAGG |
| r-1t18fl1 | TGCATCTGCTGCAAGGCGATTAAGGGTACCGA |
| r-1t18fl2 | CGCCAGGGGTGTAGATGGGCGCATAATGTGAG |
| r-1t18fl3 | TCACGTTGTTTTCCCAGTCACGAATGCCTG |
| r-1t20fl1 | GCTCGAATTCGGGAAACCTGTCGTACAGCTGA |
| r-1t20fl2 | CATTAATGCTCTAGAGGATCCCCGTTGGGTAA |
| r-1t20fl3 | CAGGTCGAAATCGGCCAACGCGCGTGGTTT |
| r-1t22fl1 | TTGCCCTTTCCACTATTAAAGAACGCCGTAAA |
| r-1t22fl2 | CAACGTCAACCAGTGAGACGGGCAGCCAGCTG |
| r-1t22fl3 | TTCTTTTCAAGGGCGAAAAACCGTCACCCA |
| r-1t24bl2 | GCACTAAATCGGAACCCTAAAGGAGTTTGGA |
| r-1t24bl3 | AATCAAGTTTTTTGGGGTCGAGGTGTGGACTC |
| r-1t2fl1 | ATGGGATTTCGCCCACGCATAACCGCAACGGC |
| r-1t2fl2 | CGGTCGCTGACGTTAGTAAATGAAACAAACTA |
| r-1t2fl3 | TCTTTCCAGAGGCTTGCAGGGAGAGCAGCG |
| r-1t4fl1 | TACAGAGGGGAGATTTGTATCATCACTTTGAA |
| r-1t4fl2 | AATTGTGTCATCGGAACGAGGGTAGATATATT |
| r-1t4fl3 | AAAGACAGCGAAATCCGCGACCTACGGTCA |
| r-1t6fl1 | AGAGGACACTTGAGATGGTTTAATAACGAACT |
| r-1t6fl2 | TAATCATTGGAACCGAACTGACCAGCCTGATA |
| r-1t6fl3 | ATCATAAGGTGAATTACCTTATGGGACGTT |
| r-1t8fl1 | AACGGAACCAAAAGAAGTTTTGCCAGTTCAGA |
| r-1t8fl2 | TAATAGTAAAATCTACGTTAATAATTCAACTT |
| r-1t8fl3 | GGGAAGAAAAATGTTTAGACTGGATTCATT |
| rt-rem1 | AGCACGTATAACGTGCTTTCCTCGTTAG |
| rt-rem2 | ACAGGGCGCGTACTATGGTTGCTTTGACG |
| rt-rem3 | ACCACACCCGCCGCGCTTAATGCGCCGCT |
| rt-rem4 | CAAGTGTAGCGGTCACGCTGCGCGTAACC |
| rt-rem5 | AGCGAAAGGAGCGGGCGCTAGGGCGCTGG |
|  |  |
| **Edge staple strands that bind to Cy3-labeled oligo3 (complementary sequence to oligo3 in lowercase)** | |
| oligo3-TT-Cy3 | agctgcaggctcgacctgcgTT/3Cy3Sp/ |
| r1t0_edge_r_2_RC_oligo3 | TTTTTTTAGACTCCTCAAGATGAAAGTATTAAGcgcaggtcgagcctgcagct |
| r1t2_edge_r_2 _RC_oligo3 | TTTTTTTCCAGAACCACCACACCACCCTCAGAGcgcaggtcgagcctgcagct |
| r1t4_edge_r_2 _RC_oligo3 | TTTTTTTGCAAAATCACCAGCATTTGGGAATTAcgcaggtcgagcctgcagct |
| r1t6_edge_r_2 _RC_oligo3 | TTTTTTTTAATAACGGAATAGGAAACCGAGGAAcgcaggtcgagcctgcagct |
| r1t8_edge_r_2 _RC_oligo3 | TTTTTTTTATTATTTATCCCGTTACAAAATAAAcgcaggtcgagcctgcagct |
| r1t10_edge_r_2_RC_oligo3 | TTTTTTTTCTTTCCTTATCAACCAATCAATAATcgcaggtcgagcctgcagct |
| r1t12_edge_r_2_RC_oligo3 | TTTTTTTTATACAAATTCTTTGTTTAGTATCATcgcaggtcgagcctgcagct |
| r1t14_edge_r_2_RC_oligo3 | TTTTTTTAGATTAAGACGCTGAAAACATAGCGAcgcaggtcgagcctgcagct |
| r1t16_edge_r_2_RC_oligo3 | TTTTTTTAGATTTTCAGGTTAGAAATAAAGAAAcgcaggtcgagcctgcagct |
| r1t18_edge_r_2_RC_oligo3 | TTTTTTTTCAATAGATAATAAACTAATAGATTAcgcaggtcgagcctgcagct |
| r1t20_edge_r_2_RC_oligo3 | TTTTTTTTTAGTCTTTAATGCAATATTTTTGAAcgcaggtcgagcctgcagct |
| r1t22_edge_r_2_RC_oligo3 | TTTTTTTTACTTCTTTGATTAAATTAACCGTTGcgcaggtcgagcctgcagct |
| r-­‐1t2_edge_l_2_RC_oligo3 | TTTTTTTAACGATCTAAAGTCAGCCCTCATAGTcgcaggtcgagcctgcagct |
| r-­‐1t4_edge_l_2_RC_oligo3 | TTTTTTTGATCGTCACCCTCTTAAAGGCCGCTTcgcaggtcgagcctgcagct |
| r-­‐1t6_edge_l_2_RC_oligo3 | TTTTTTTGAACGAGGCGCAGGCTCCATGTTACTcgcaggtcgagcctgcagct |
| r-­‐1t8_edge_l_2_RC_oligo3 | TTTTTTTATTATACCAGTCACGATTTTAAGAACcgcaggtcgagcctgcagct |
| r-­‐1t10_edge_l_2_RC_oligo3 | TTTTTTTAATCGTCATAAATATAGCGTCCAATAcgcaggtcgagcctgcagct |
| r-­‐1t12_edge_l_2_RC_oligo3 | TTTTTTTAATATAATGCTGTGCTTAGAGCTTAAcgcaggtcgagcctgcagct |
| r-­‐1t14_edge_l_2_RC_oligo3 | TTTTTTTAAAGCTAAATCGGCAATAAAGCCTCA cgcaggtcgagcctgcagct |
| r-­‐1t16_edge_l_2_RC_oligo3 | TTTTTTTACAAGAGAATCGAGCCTGAGAGTCTGcgcaggtcgagcctgcagct |
| r-­‐1t18_edge_l_2_RC_oligo3 | TTTTTTTATTGACCGTAATGCCGTGGGAACAAAcgcaggtcgagcctgcagct |
| r-­‐1t20_edge_l_2_RC_oligo3 | TTTTTTTGTGCCAAGCTTGCCGTTGTAAAACGAcgcaggtcgagcctgcagct |
| r-­‐1t22_edge_l_2_RC_oligo3 | TTTTTTTATTGGGCGCCAGGGGGGAGAGGCGGTcgcaggtcgagcctgcagct |
| r-­‐1t24_edge_l_2_RC_oligo3 | TTTTTTTACTACGTGAACCATCTATCAGGGCGAcgcaggtcgagcctgcagct |
|  |  |
| **Core staples that bind to oligo1-myosin V or VI** |  |
| BG-oligo1 | TTTTTTgatacgcgccaatctctata |
| r0t1mr_fr-TR-oligo1 | CCGTATATGGCCTTGATATTCAGAGCCACCtatagagattggcgcgtatc |
| r1t12fr2-MR-oligo1 | tatagagattggcgcgtatcTCCTAATTACGCTCAACAGTAGGGAACACCGG |
| r0t17mr_fr-BR-oligo1 | CTGAATACGTATTAAATCCTTTGGCAAATCtatagagattggcgcgtatc |
| r0t17ml3_hp_org-BL-oligo1 | CAGCTTTCCTATTACGCCAGCTGGTAGCTGTTtatagagattggcgcgtatc |
| r-1t12fl1-ML-oligo1 | tatagagattggcgcgtatcCTGGAAGTACATCCAATAAATCATTTTTGCGG |
| r0t1ml3-TL-oligo1 | TTTTCACGCCGATAGTTGCGCCGAACTTTTTCtatagagattggcgcgtatc |
| **Core staple strands that bind to oligo5-myosin V or VI** |  |
| BG-oligo5 | TTTTTTcgttattacttagcatttgg |
| r0t1mr_fr-TR-oligo5 | CCGTATATGGCCTTGATATTCAGAGCCACCccaaatgctaagtaataacg |
| r1t12fr2-MR-oligo5 | ccaaatgctaagtaataacgTCCTAATTACGCTCAACAGTAGGGAACACCGG |
| r0t17mr_fr-BR-oligo5 | CTGAATACGTATTAAATCCTTTGGCAAATCccaaatgctaagtaataacg |
| r0t17ml3_hp_org-BL-oligo5 | CAGCTTTCCTATTACGCCAGCTGGTAGCTGTTccaaatgctaagtaataacg |
| r-1t12fl1-ML-oligo5 | ccaaatgctaagtaataacgCTGGAAGTACATCCAATAAATCATTTTTGCGG |
| r0t1ml3-TL-oligo5 | TTTTCACGCCGATAGTTGCGCCGAACTTTTTCccaaatgctaagtaataacg |
| **Core staple strands that bind to photocleavable oligo5-myosin V or VI** |  |
| BG-oligo4PCRColigo5 | cctaccctctgatgtcttcga /iSpPC/ accaaatgctaagtaataacg |
|  |  |
| r0t1mr_fr-TR-oligo4 | CCGTATATGGCCTTGATATTCAGAGCCACCtcgaagacatcagagggtagg |
| r1t12fr2-MR-oligo4 | tcgaagacatcagagggtaggTCCTAATTACGCTCAACAGTAGGGAACACCGG |
| r0t17mr_fr-BR-oligo4 | CTGAATACGTATTAAATCCTTTGGCAAATCtcgaagacatcagagggtagg |
| r0t17ml3_hp_org-BL-oligo4 | CAGCTTTCCTATTACGCCAGCTGGTAGCTGTTtcgaagacatcagagggtagg |
| r-1t12fl1-ML-oligo4 | tcgaagacatcagagggtaggCTGGAAGTACATCCAATAAATCATTTTTGCGG |
| r0t1ml3-TL-oligo4 | TTTTCACGCCGATAGTTGCGCCGAACTTTTTCtcgaagacatcagagggtagg |
| **Core staple strands that do not bind to either oligo 1 or oligo2 (control)** | **)** |
| r0t1mr_fr-TR | CCGTATATGGCCTTGATATTCAGAGCCACC |
| r1t12fr2-MR | TCCTAATTACGCTCAACAGTAGGGAACACCGG |
| r0t17mr_fr-BR | CTGAATACGTATTAAATCCTTTGGCAAATC |
| r0t17ml3_hp_org-BL | CAGCTTTCCTATTACGCCAGCTGGTAGCTGTT |
| r-1t12fl1-ML | CTGGAAGTACATCCAATAAATCATTTTTGCGG |
| r0t1ml3-TL | TTTTCACGCCGATAGTTGCGCCGAACTTTTTC |
| **Purification strands** |  |
| spacer-strand | CGATGGATGACTGACTGATGGATGACTTAAATTGACTATGACTATGATACTGACTGATTACG |
| biotin-strand | CATCCATCAGTCAGTCATCCATCGTTTTTTT-biotin |
| origami-strand (r1t0) | TTTTTTTAGACTCCTCAAGATGAAAGTATTAAGTTGGATAGTCAGTATCATAGTCATAGTCAA |
| elution-strand  blocking oligos | CGTAATCAGTCAGTATCATAGTCATAGTCAATTTAAGTCATCCATCAGTCAGTCATCCATCG  NNNNNNNNNN NNNNNNNNNN NNNNNNNNNN NNNNNNNNNN NN |
