# Supplementary material for: Tuning myosin-driven sorting on cellular actin networks
Source: eLife. 2015 Mar 4;4:e05472. doi: 10.7554/eLife.05472 (PMC4377546; doi:10.7554/eLife.05472)
Supplement: Supplementary file 2. — Summary of run length measurements. DOI: http://dx.doi.org/10.7554/eLife.05472.016 [file elife05472s002.docx]

**Supplementary file 2 ­– Summary of run length data.**

Mean run lengths for indicated experimental conditions. The minimum number of detected trajectories was 112 and on average 852. For each experimental condition, data were collected on at least 3 different keratocytes.

Analysis of myosin labeled scaffold movement was restricted to scaffolds that appeared for more than 6 continuous frames (3 sec) and covered a distance of more than 3 pixels (320 nm). Run lengths were measured by fitting a trajectory into linear segments of 300 nm starting with the first appearance of the scaffold. Run-length distribution was fitted to the truncated cumulative distributive function (CDF) of a single exponential distribution, CDF(*x*) = 1–e^–(^*^x^*^–^*^xmin^*^)/^*^λ^*, where *λ* is the mean run length and *x_min_* is the minimum measurable run length (300 nm). End-to-end speeds were calculated by dividing measured run length by the total time the scaffold remained bound to the acitn filaments or keratocyte network. The run length is reported as mean ± S.E.M. of the parameter *λ*, derived from the fit. The S.E.M. was estimated by the bootstrap method.

|  |  | **〈*RL*〉 (µm)** | | |
| --- | --- | --- | --- | --- |
| **Experiments** | **Track** | **to cell periphery (+ end)** | **to cell center (– end)** | **N** |
| 1V:0VI | keratocyte | 0.495 ± 0.063 | – | 413 |
| 2V:0VI | actin filament | 1.12 ± 0.18 | – | 287 |
| 2V:0VI | keratocyte | 0.732 ± 0.018 | – | 1688 |
| 3V:0VI | keratocyte | 0.831 ± 0.029 | – | 895 |
| 4V:0VI | keratocyte | 1.14 ± 0.06 | – | 385 |
| 5V:0VI | keratocyte | 1.43 ± 0.06 | – | 776 |
| 6V:0VI | keratocyte | 1.84 ± 0.06 | – | 1398 |
| 0V:1VI | keratocyte | – | 0.388 ± 0.031 | 317 |
| 0V:2VI | actin filament | – | 0.908 ± 0.103 | 222 |
| 0V:2VI | keratocyte | – | 0.696 ± 0.016 | 1897 |
| 0V:3VI | keratocyte | – | 0.990 ± 0.010 | 205 |
| 0V:4VI | keratocyte | – | 1.19 ± 0.12 | 112 |
| 0V:5VI | keratocyte | – | 1.43 ± 0.03 | 687 |
| 0V:6VI | keratocyte | – | 2.14 ± 0.06 | 1185 |
| 1V:1VI | actin filament | 0.767 ± 0.061 | 0.588 ± 0.045 | 202 |
| 1V:1VI | keratocyte | 0.644 ± 0.032 | 0.628 ± 0.031 | 828 |
| 1V:2VI | keratocyte | 0.612 ± 0.030 | 0.651 ± 0.023 | 993 |
| 1V:3VI | keratocyte | 0.688 ± 0.045 | 0.994 ± 0.037 | 1117 |
| 1V:5VI | keratocyte | 0.558 ± 0.037 | 1.24 ± 0.04 | 1011 |
| 2V:1VI | keratocyte | 0.785 ± 0.048 | 0.672 ± 0.039 | 574 |
| 2V:2VI | keratocyte | 0.616 ± 0.031 | 0.790 ± 0.037 | 1837 |
| 2V:3VI | keratocyte | 0.469 ± 0.029 | 0.776 ± 0.042 | 480 |
| 2V:4VI | keratocyte | 0.854 ± 0.038 | 0.966 ± 0.034 | 1161 |
| 3V:1VI | keratocyte | 0.841 ± 0.030 | 0.555 ± 0.036 | 1348 |
| 3V:2VI | keratocyte | 0.912 ± 0.042 | 0.843 ± 0.066 | 620 |
| 3V:3VI | keratocyte | 0.817 ± 0.065 | 0.926 ± 0.110 | 266 |
| 4V:2VI | keratocyte | 0.866 ± 0.028 | 0.568 ± 0.026 | 1271 |
| 5V:1VI | keratocyte | 1.19 ± 0.05 | 0.547 ±0.078 | 526 |
